# Building a semantic web-based metadata repository for facilitating detailed clinical modeling in cancer genome studies

**DOI:** 10.1186/s13326-017-0130-4

**Published:** 2017-06-05

**Authors:** Deepak K. Sharma, Harold R. Solbrig, Cui Tao, Chunhua Weng, Christopher G. Chute, Guoqian Jiang

**Affiliations:** 1 Department of Health Sciences Research, Mayo Clinic, 200 First St SW, Rochester, MN 55905 USA; 2 University of Texas Health Science Center at Houston, Houston, TX USA; 3 Columbia University, New York, NY USA; 4 Johns Hopkins University, Baltimore, MD USA

**Keywords:** Detailed Clinical Models (DCMs), Clinical Information Modeling Initiative (CIMI), Common Data Elements (CDEs), The Cancer Genome Atlas (TCGA), Cancer Studies, Semantic Web Technologies

## Abstract

**Background:**

Detailed Clinical Models (DCMs) have been regarded as the basis for retaining computable meaning when data are exchanged between heterogeneous computer systems. To better support clinical cancer data capturing and reporting, there is an emerging need to develop informatics solutions for standards-based clinical models in cancer study domains. The objective of the study is to develop and evaluate a cancer genome study metadata management system that serves as a key infrastructure in supporting clinical information modeling in cancer genome study domains.

**Methods:**

We leveraged a Semantic Web-based metadata repository enhanced with both ISO11179 metadata standard and Clinical Information Modeling Initiative (CIMI) Reference Model. We used the common data elements (CDEs) defined in The Cancer Genome Atlas (TCGA) data dictionary, and extracted the metadata of the CDEs using the NCI Cancer Data Standards Repository (caDSR) CDE dataset rendered in the Resource Description Framework (RDF). The ITEM/ITEM_GROUP pattern defined in the latest CIMI Reference Model is used to represent reusable model elements (mini-Archetypes).

**Results:**

We produced a metadata repository with 38 clinical cancer genome study domains, comprising a rich collection of mini-Archetype pattern instances. We performed a case study of the domain “clinical pharmaceutical” in the TCGA data dictionary and demonstrated enriched data elements in the metadata repository are very useful in support of building detailed clinical models.

**Conclusion:**

Our informatics approach leveraging Semantic Web technologies provides an effective way to build a CIMI-compliant metadata repository that would facilitate the detailed clinical modeling to support use cases beyond TCGA in clinical cancer study domains.

## Background

Detailed Clinical Models (DCMs) have been regarded as the basis for retaining computable meaning when data are exchanged between heterogeneous computer systems [1]. Several independent clinical information modeling initiatives have emerged, including Health Level 7 (HL7) Detailed Clinical Models (DCM) [2], ISO/CEN EN13606/Open-EHR Archetype [3], Intermountain Healthcare Clinical Element Models (CEMs) [4], and the Clinical Information Model in the Netherlands [5]. The collective clinical information modeling community has recently initiated an international collaboration effort known as the Clinical Information Modeling Initiative (CIMI) [6]. The primary goal of CIMI is to provide a shared repository of detailed clinical information models based on common formalism.

While the primary focus of these modeling efforts has been on interoperability between electronic health record (EHR) systems, there are also emerging interests in the use of detailed clinical models in the context of clinical research and broad secondary use of EHR data. A typical use case is the Office of the National Coordinator (ONC) Strategic Health IT Advanced Research Projects Area 4 (SHARPn) [7, 8], in which the Intermountain Healthcare CEMs have been adopted for normalizing patient data for the purpose of secondary use. In the context of clinical research, for example, Clinical Data Interchange Standards Consortium (CDISC) intends to build reusable domain-specific templates under its SHARE project [9, 10].

To better support clinical cancer data capturing and reporting, there is an emerging need to develop informatics solutions for standards-based clinical models in clinical cancer study domains. For example, National Cancer Institute (NCI) has implemented the Cancer Data Standards Repository (caDSR) [11], together with a controlled terminology service (known as Enterprise Vocabulary Services – EVS), as the infrastructure to support a variety of use cases from different clinical cancer study domains. NCI caDSR has adopted the ISO 11179 metadata standard that specifies a standard data structure for a common data element (CDE) [12, 13].

The use case in this study is based on The Cancer Genome Atlas (TCGA) Biospecimen Core Resource (BCR) data dictionary [14]. The data dictionary is used to create clinical data collection forms for different clinical cancer genome study domains. TCGA clinical data include vital status at time of report, disease-specific diagnostic information, initial treatment regiments and participant follow-up information [15]. The data dictionary groups a preferred set of CDEs per TCGA cancer study domain and renders them as an XML Schema document. All clinical data collected are validated against these schemas, which provides a layer of standards-based data quality control. All the CDEs are recorded in the NCI caDSR repository, the implementation of which is based on the ISO 11179 standard. We envision that cataloging a preferred set of CDEs for each clinical cancer study domain is analogous to identifying or creating preferred Detailed Clinical Models for a given domain.

The objective of the study is to develop and evaluate a cancer genome study metadata management system that serves as a key infrastructure in supporting clinical information modeling in cancer genome study domains. We leveraged a Semantic Web-based metadata repository enhanced with both the ISO11179 metadata standard and the Clinical Information Modeling Initiative (CIMI) Reference Model (RM). We used the CIMI-compliant archetype patterns to represent preferred set of CDEs used in the TCGA data dictionary and identified additional data elements from caDSR for a given domain. And then we loaded a RDF-metadata repository with data elements based on these archetype patterns. We hypothesize that clinical information modeling tools can leverage such metadata repository to reuse data elements already widely adopted by clinical genomic research studies (e.g., TCGA studies).

## Methods

### Materials

#### ISO 11179 and its OWL representations

ISO 11179 is an international standard known as the ISO/IEC 11179 Metadata Registry (MDR) standard [12]. It consists of six parts. Part 3 of the standard uses a meta-model to describe the information modeling of a metadata registry, which provides a mechanism for understanding the precise structure and components of domain-specific models.

**Fig. 1:**
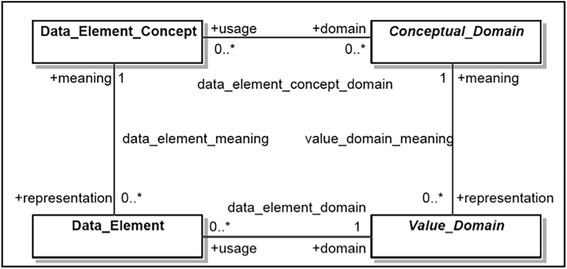
High-level data description meta-model in ISO 11179 specification

Figure 1 shows a diagram illustrating the high-level data description meta-model in the ISO 11179 specification. The Data Element is one of the foundational concepts in the specification. ISO 11179 also specifies the relationships and interfaces between data elements, value sets (i.e., enumerated value domains) and standard terminologies.

Several Semantic Web-based representations of the ISO 11179 Part 3 meta-model have been created for projects including the XMDR project [16], Semantic MDR in a European SALUS project [17] and CDISC2RDF in FDA PhUSE Semantic Technology project [18]. In the present study, we utilize a meta-model schema in OWL/RDF developed in the CDISC2RDF project, which is a subset of ISO 11179 Part 3 meta-model.

#### Reference model in UML

The CIMI Reference Model (RM) is an information model from which CIMI’s clinical models (i.e., archetypes) are derived [6]. The CIMI DCM’s are expressed as formal constraints on the underlying RM. The CIMI Reference Model is represented in the Unified Modeling Language (UML). The September 5, 2014 version of the CIMI Reference Model (v2.0.1) had four packages: 1) CIMI Core Model; 2) Data Value Types; 3) Primitive Types and 4) Party. While the core CIMI Reference Model Classes are defined in the CIMI Core Model package, the Party package defines the generic concepts of PARTY, ROLE and related details for describing potential demographic attributes. Both of these packages utilize the types declared in the Data Value Types and Primitive Types packages.

**Fig. 2:**
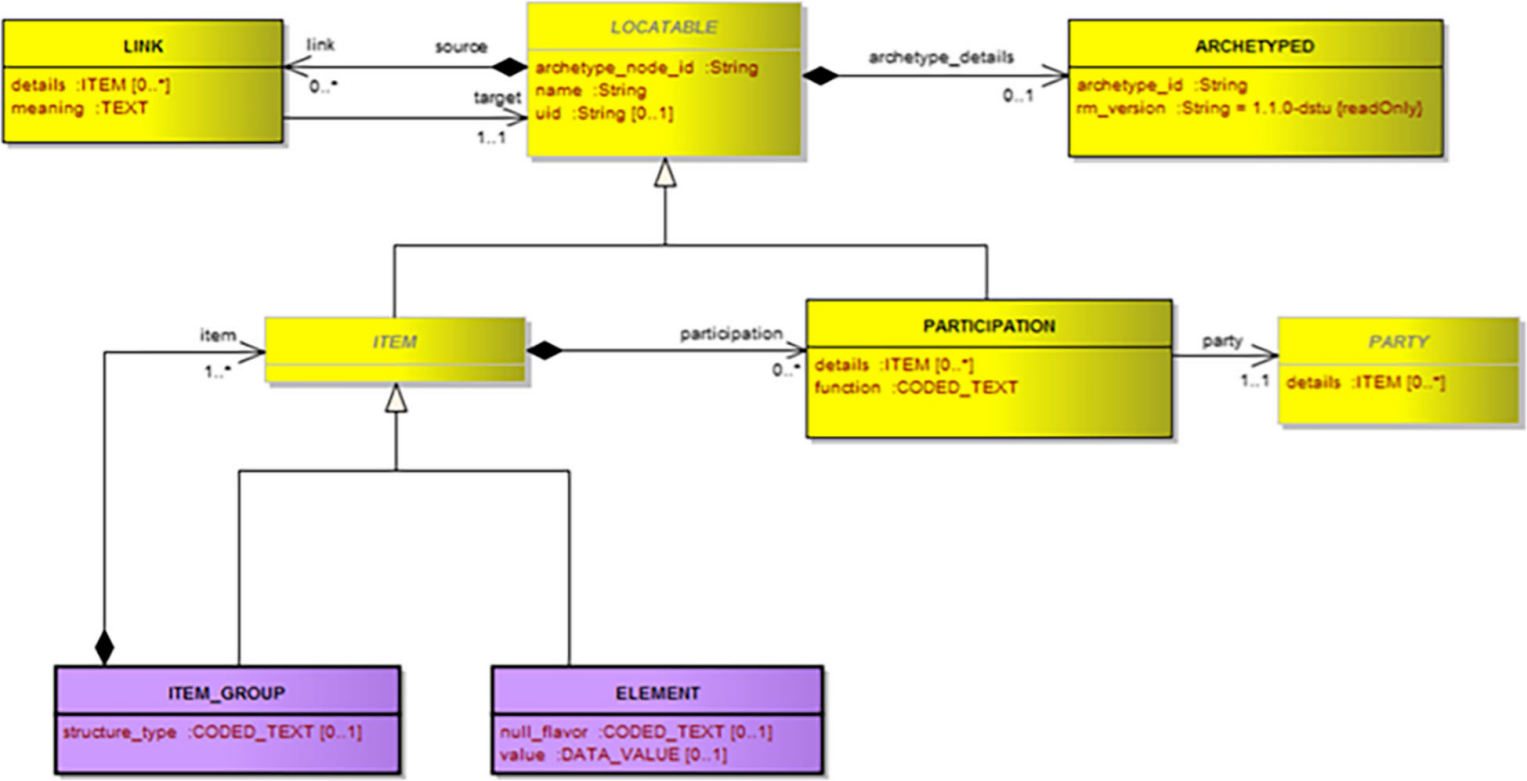
CIMI Core Model in UML Diagram

**Fig. 3:**
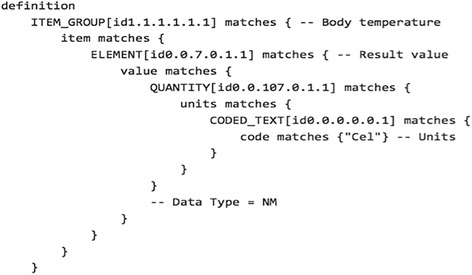
The definition section of an archetype for a CIMI “Body temperature” concept. The definition is rendered in archetype definition language (ADL)

Figure 2 shows partial view of UML Class diagram of the CIMI Core Model. The classes ITEM, ITEM_GROUP, and ELEMENT form very generic pattern (referred as ‘ITEM/ITEM_GROUP Pattern’ here onwards) that can be used recursively to represent almost any clinical information. The ITEM_GROUP class represents the grouping variant of ITEM as an ordered list whereas the ELEMENT class represents a “leaf” ITEM which carries no further recursion. Figure 3 shows Archetype Definition Language (ADL) [19] definition of a “Body Temperature” archetype, which illustrates how ITEM_GROUP and ELEMENT can be combined when representing a clinical concept.

#### The caDSR CDE dataset

NCI caDSR is part of the NCI Cancer Common Ontological Representation Environment (caCORE) infrastructure and uses caCORE resources to support data standardization in cancer clinical research studies [11]. The system includes an administrator web interface for overall system and CDE management activities. Integrated with caCORE Enterprise Vocabulary Services (EVS), the CDE Curation Tool aids developers in consumption of NCI controlled vocabulary and standard terminologies for naming and defining CDEs.

**Fig. 4:**
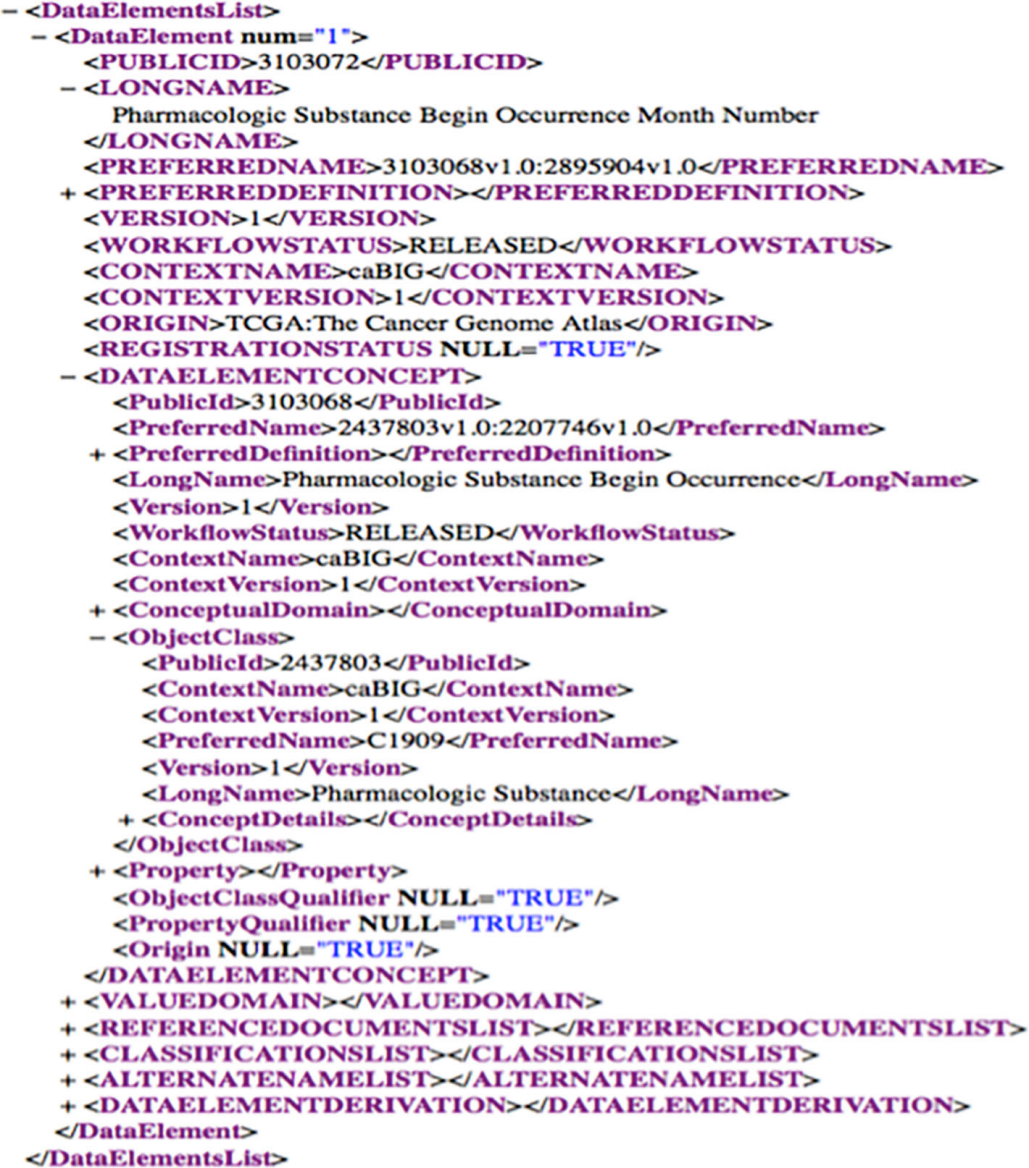
The CDE “Pharmacologic Substance Begin Occurrence Month Number” in XML recorded in the NCI caDSR

NCI caDSR provides the ability to download CDEs in either Excel or XML format [20], which we used to download an XML image of all non-retired production CDEs (i.e., CDEs with Workflow status NOT = “RETIRED”) as of August 7, 2014. Figure 4 shows an XML rendering of the CDE “Pharmacologic Substance Begin Occurrence Month Number” from the NCI caDSR.

#### The TCGA data dictionary

**Fig. 5:**
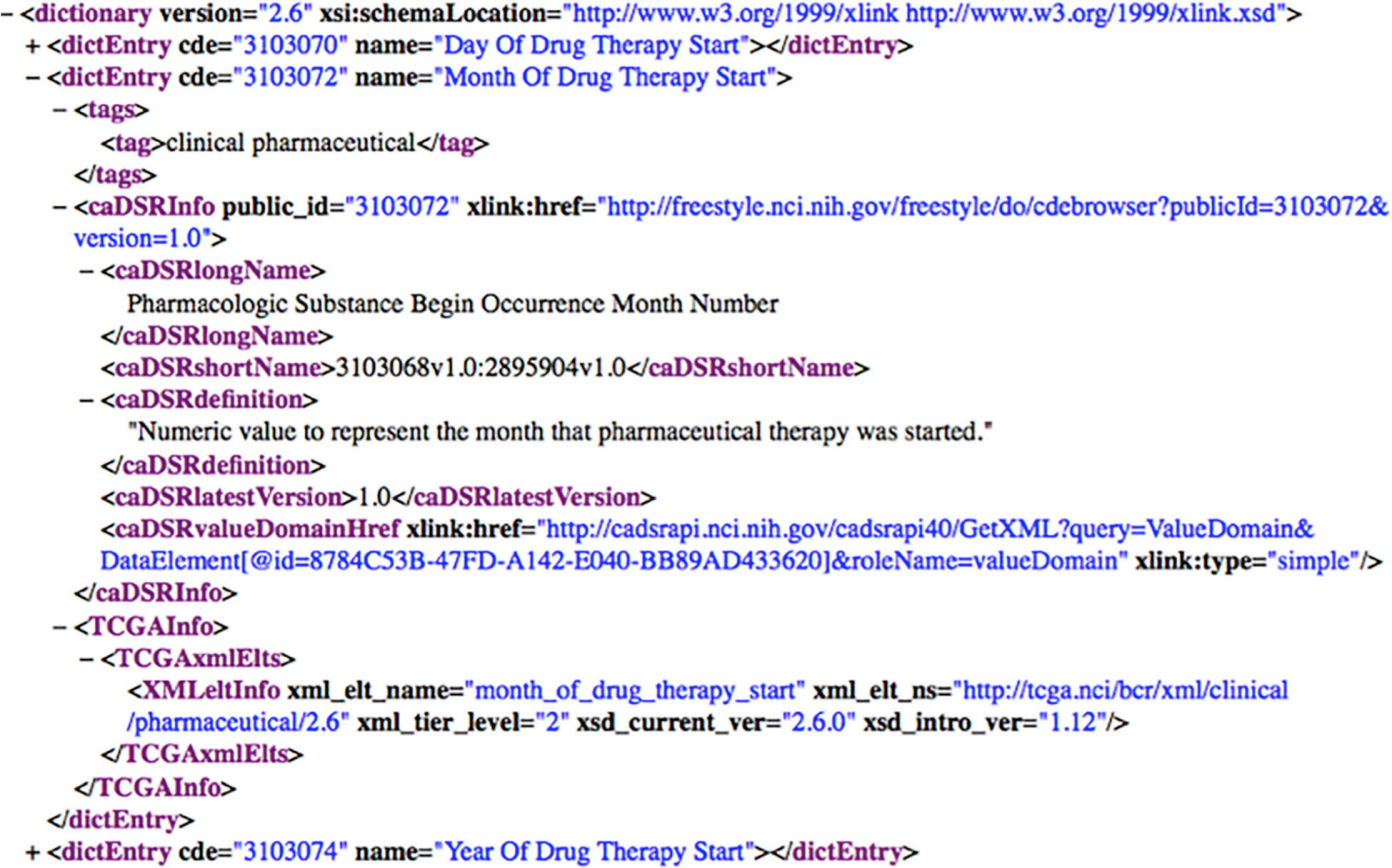
A TCGA data dictionary variable ‘Month Of Drug Therapy Start’ annotated with the CDE “Pharmacologic Substance Begin Occurrence Month Number” that is originally recorded in the NCI caDSR

The Cancer Genome Atlas (TCGA), a joint venture supported by the NCI and the National Human Genome Research Institute (NHGRI), is a comprehensive and coordinated effort to accelerate the understanding of the molecular basis of cancer through the application of genome analysis technologies, including large-scale genome sequencing. Being a component of TCGA Research Network, the Biospecimen Core Resource (BCR) serves as the centralized tissue processing and clinical data collection center. A BCR data dictionary has been produced using the standard CDEs from NCI caDSR. The CDEs in the data dictionary are publicly available in the XML format. In this project, we will download a snapshot of the data dictionary from the TCGA website [14]. Figure 5 shows a TCGA data dictionary variable ‘Month Of Drug Therapy Start’ is annotated with the CDE “Pharmacologic Substance Begin Occurrence Month Number” from the NCI caDSR.

### Methods

**Fig. 6:**
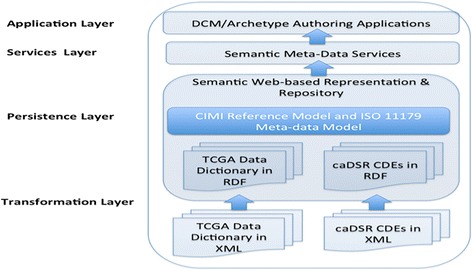
System architecture of our proposed approach

Figure 6 shows the system architecture of our proposed approach. The system comprises four layers: a RDF transformation layer; a RDF store-based persistence layer; a semantic services layer and an authoring application layer. This paper focuses on transformation layer and persistence layer.

#### RDF transformation of caDSR and TCGA datasets

The XML2RDF tool, developed by the Redefer project [21], was used to transform the XML-based TCGA data dictionary and the XML-based caDSR production CDEs into a corresponding RDF representation. We loaded the resulting RDF datasets into a 4store instance, an open-source RDF triple-store and exposed them via a SPARQL endpoint, allowing us to use the SPARQL query language to preform semantic queries across the datasets.

#### OWL-based schema for CIMI Reference Model and ISO 11179

We used the latest version of CIMI Reference Model (v2.0.1) in the XML Metadata Interchange (XMI) format. We then converted the CIMI Reference Model from XMI to RDF format using the Redefer XML2RDF transformation services [21]. We then defined the SPARQL queries to retrieve the UML based elements of the CIMI Reference Model such as classes, attributes and associations. We created a JAVA program that produces an OWL rendering of the CIMI Reference Model using the UML2OWL mappings specified by the Object Management Group (OMG) Ontology Definition meta-model (ODM) standard [22]. We finally harmonized and created an OWL-based schema for CIMI Reference Model and ISO11179.

#### Defining and populating reusable archetype patterns

**Fig. 7:**
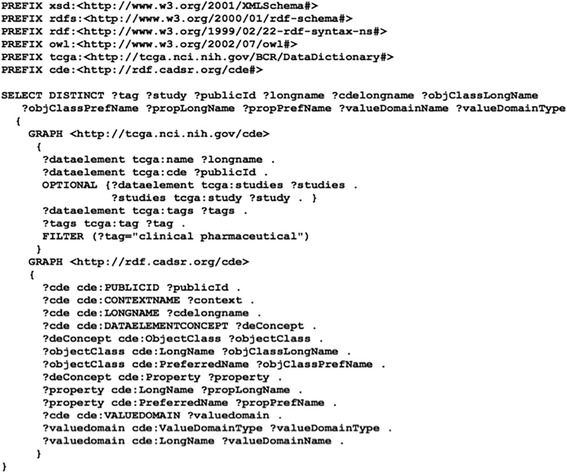
A SPARQL query example that retrieves all CDEs of the domain “clinical pharmaceutical”

We defined reusable archetype patterns that capture the clinical cancer domains defined in the TCGA data dictionary, their associated CDEs and the metadata structures (Object Class, Property, Value Domain, etc.) recorded in the caDSR data repository. We then defined a collection of SPARQL queries to retrieve the metadata elements from both the TCGA data dictionary and the caDSR CDE dataset. Figure 7 shows a SPARQL query example that retrieves all CDEs of the domain “clinical pharmaceutical” defined in the TCGA data dictionary and their metadata recorded in caDSR CDE dataset. We also developed a JAVA program that populates all reusable archetype patterns in TCGA clinical cancer domains into the instance data using the OWL-based schema that we created.

#### Evaluation of clinical utility

We performed a case study for the domain Clinical Pharmaceutical to demonstrate clinical utility of our approach. Specifically, we demonstrated how many properties and enumerated value domains are enriched for the domain through the ISO 11179-based data elements recorded in the NCI caDSR. We then evaluate clinical utility of the enriched data elements using a Medication template defined in CDISC Clinical Data Acquisition Standards Harmonization (CDASH) standard [23]. We created the alignment between the CDISC Medication template and the CDEs retrieved from the domain Clinical Pharmaceutical and the alignment consensus was achieved through a series of discussions among the project team members.

## Results

In total, the TCGA data dictionary contains 38 clinical cancer domains and 775 CDEs, which covers 21 cancer types. Table 1 shows a list of examples showing the clinical cancer domains and the number of CDEs in each domain.

**Table 1:** A list of examples showing TCGA clinical cancer study domains

Clinical Cancer Domains	Number of CDEs	Notes
clinical shared	98	
clinical laml	49	Acute Myeloid Leukemia [LAML]
clinical cesc	47	Cervical squamous cell carcinoma and endocervical adenocarcinoma [CESC]
clinical lgg	33	Brain Lower Grade Glioma [LGG]
clinical lihc	31	Liver hepatocellular carcinoma [LIHC]
clinical prad	25	Prostate adenocarcinoma [PRAD]
clinical paad	23	Pancreatic adenocarcinoma [PAAD]
clinical thca	20	Thyroid carcinoma [THCA]
clinical shared stage	19	
clinical pharmaceutical	18	

**Fig. 8:**
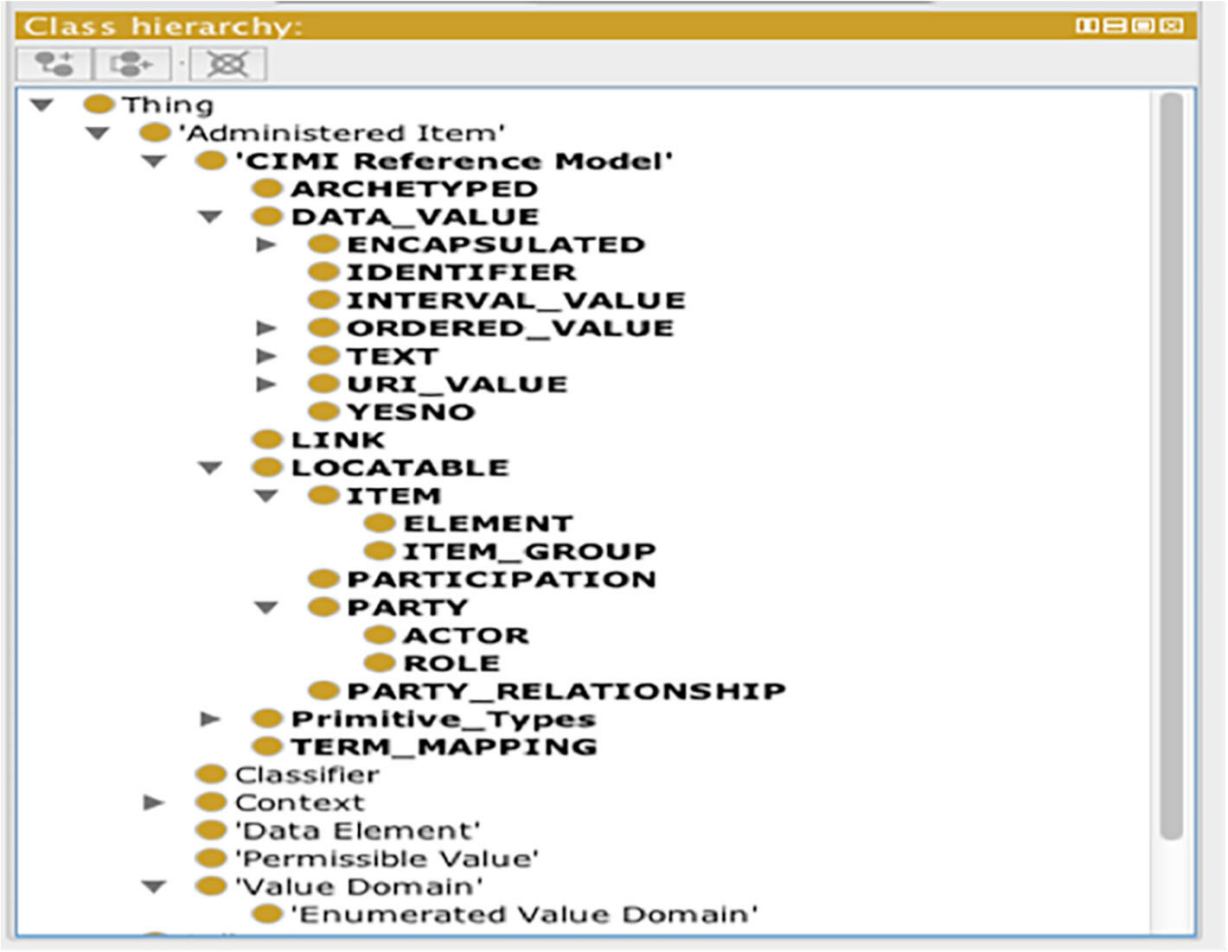
A screenshot of Protégé 4 environment showing an OWL-based schema. The schema is for a CIMI Reference Model harmonized with ISO 11179 model

We created an OWL rendering of CIMI Reference Model and harmonized it with the ISO 11179 metadata model schema, in which all classes defined in the CIMI Reference Model are asserted as the subclasses of an ISO 11179 class *mms:AdministeredItem*. Figure 8 shows a screenshot of Protégé 4 environment illustrating the class hierarchy of OWL-based schema for harmonized CIMI Reference Model with ISO 11179 model.

We populated reusable archetype patterns against the OWL-based schema and produced a metadata repository based in RDF format. The repository covers all 38 clinical cancer study domains, comprising 316 distinct object classes, 4719 distinct properties, 1015 non-enumerated value domains and 1795 enumerated value domains (i.e., value sets).

Table 2 shows two pattern examples extracted from the TCGA domain “clinical pharmaceutical”. Pattern 1 captures a number of CDEs asserted in the TCGA data dictionary; Pattern 2 captures equivalent metadata structures (Object Class, Property, Value Domain, etc.) recorded in the caDSR data repository. The 7 CDEs captured in Pattern 1 have their “Object Class” in common that is “Pharmacologic Substance.” The “Pharmacologic Substance” is linked with three “Property” instances: “Begin Occurrence,” “End Occurrence” and “Continue Occurrence.” The properties are associated with 4 Value Domains: “Event Year Number”, “Event Month Number,” “Event Day Number”, and “Yes No Character Indicator”.

**Table 2:** Two pattern examples extracted from the TCGA domain “clinical pharmaceutical”

Pattern 1	Pattern 2
clinical pharmaceutical [ITEM_GROUP]	clinical pharmaceutical [ITEM_GROUP]
	Pharmacologic Substance [ITEM_GROUP]
Year Of Drug Therapy Start [ELEMENT]	Begin Occurrence [ITEM_GROUP]
Month Of Drug Therapy Start [ELEMENT]	Event Year Number [ELEMENT]
Day Of Drug Therapy Start [ELEMENT]	Event Month Number [ELEMENT]
	Event Day Number [ELEMENT]
Year Of Drug Therapy End [ELEMENT]	End Occurrence [ITEM_GROUP]
Month Of Drug Therapy End [ELEMENT]	Event Year Number [ELEMENT]
Day Of Drug Therapy End [ELEMENT]	Event Month Number [ELEMENT]
	Event Day Number [ELEMENT]
Therapy Ongoing [ELEMENT]	Continue Occurrence [ITEM_GROUP]
	Yes No Character Indicator [ELEMENT]

### Evaluation results

**Fig. 9:**
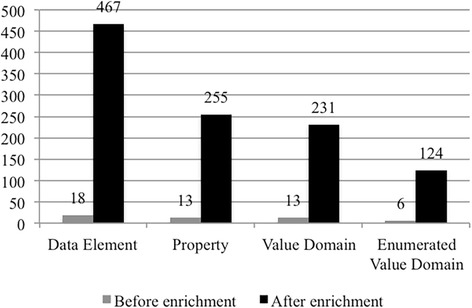
A bar graph showing the enrichment for the domain Clinical Pharmaceutical. The enrichment by data element, property, value domain and enumerated value domain is illustrated

As a case study, we looked into the domain Clinical Pharmaceutical that contains 18 CDEs. We retrieved the object classes recorded in caDSR and identified 11 distinct object classes. And then, we retrieved globally in the caSDR CDE datasets for all properties and value domains associated with the 11 object classes. Figure 9 shows a bar graph illustrating the enrichment for the domain Clinical Pharmaceutical by data element, property, value domain and enumerated value domain. The graph indicated that the domain is greatly enriched with properties and value domains associated with those 11 object classes, which forms a pool of data elements that could be used to build detailed clinical models in this domain.

To evaluate clinical utility of our approach, we aligned the data elements between CDASH Medication and TCGA Clinical Pharmaceutical. Table 3 shows the alignment results. Out of 20 CDASH data elements with their data collection questions, 9 of them aligned with the CDEs asserted in the TCGA data dictionary whereas 10 of them aligned with those enriched data elements identified from our system. This shows that the addition of the enriched data elements can not only guide us to evaluate a data dictionary by identifying the gaps, but also provide a pool of data elements to choose from to help build clinical models. We believe that the results demonstrated that enriched data elements are useful in building a clinical model for the use cases beyond original TCGA data dictionary.

**Table 3:** Alignment results of data elements between CDASH Medication and TCGA Clinical Pharmaceutical

Question Text	Prompt Data Element Name	TCGA CDEs or Enriched Data Elements
Were any medications taken?	Any meds	***Administered***
What is the medication/treatment identifier?	CM number	***Identifier; Unique Identifier***
What was the term for the medication/therapy taken?	Medication or Therapy	Drug Name
Did the subject take < specific medication/treatment > ?	<specific medication/treatment>	***Cytokine Administered; Placebo Bevacizumab Administered; HER2/neu Administered***
What were the active ingredients?	Active Ingredients	***PubChem Compound Identifier***
For what indication was the medication/therapy taken?	Indication	***Indication***
What was the ID for the adverse events(s) for which the medication was taken?	AE ID	***Toxicity Description; Toxicity Grade***
What was the ID of the medical history condition(s) for which the medication was taken?	MH ID	
What was the individual dose of the medical/therapy?	Dose	Prescribed Dose
What was the total daily dose of the medication therapy?	Total Daily Dose	Cumulative Agent Total Dose
What was the unit of the medical/therapy?	Dose Unit	Total Dose Units;Prescribed Dose Units
What was the dose form of the medication/therapy?	Dose Form	***Pharmaceutical Dosage Form Code***
What was the frequency of the medication/therapy?	Frequency	Number Cycles
What was the route of administration of the medication/therapy?	Route	Route Of Administration
What was the start date of the medication/therapy?	Start Date	Year Of Drug Therapy Start;Month Of Drug Therapy Start; Day Of Drug Therapy Start
What was the start time of the medication/therapy?	Start Time	***Agent Administered Begin Time***
Was the medication/therapy taken prior to the study?	Taken Prior to Study?	***Prior Therapy Treatment Regimen***
What was the end date of the medication/therapy?	End Date	Year Of Drug Therapy End; Month Of Drug Therapy End; Day Of Drug Therapy End
What was the end time of the medication/therapy?	End Time	***Agent Administered End Time***
Is the medication/therapy still ongoing?	Ongoing	Therapy Ongoing

Bold italic font indicates an enriched data element

## Discussion

In this study, we first transformed the TCGA data dictionary and the caDSR CDE dataset from their XML format to the RDF-based representations. This transformation makes it easier to query caDSR metadata elements that correspond to the CDEs defined in the TCGA data dictionary. The TCGA data dictionary terminology bindings enable exploration of additional metadata associated with CDEs that would otherwise be challenging to associate programmatically. These newly discovered elements help get better insight about the gaps in their proper and efficient usage in the models that data dictionaries intend to represent. Second, the CIMI Reference Model offers a simple recursive pattern (with its ITEM, ITEM_GROUP and ELEMENT classes) to represent CDEs in each TCGA cancer genome study sub-domain, as instances. The CIMI Reference Model is transformed from its UML format to a corresponding OWL representation and harmonized it with a subset of ISO 11179 metadata model. As indicated above, the transformation of the TCGA data dictionary, caDSR CDEs, CIMI Reference Model, ISO 11179 into RDF normalizes their representation and makes it easier to query the content using a standard SPARQL end-point. Finally, we performed a case study in the domain of ‘Clinical Pharmaceutical’ and demonstrated the clinical utility of our proposed approach. We consider that this approach is novel as to our best knowledge this is the first attempt trying to reuse the CDEs recorded in the caDSR for supporting creating clinical information models based on the CIMI Reference Model.

The metadata repository system proposed in this study has the following three major implications. The first implication is that the system would enable producing a profile of CIMI-compliant detailed clinical models for TCGA clinical cancer study domains by leveraging the best practice of detailed clinical modeling in CIMI community. Pattern 1 as shown in Table 2 is designed to capture a preferred set of CDEs and metadata for each domain asserted in the TCGA data dictionary. The semantics captured in Pattern 1 should be equivalent to those asserted in the TCGA XML Schemas. In other words, Pattern 1 serves as the CIMI-compliant representation of a preferred set of CDEs in a TCGA cancer study domain.

The second implication is that we gained new insights on how the ISO 11179 standard could interact with the CIMI Reference Model for supporting detailed clinical modeling. The added value would ultimately be the ability to represent ISO 11179 based constructs as constraints on CIMI Reference Model. Pattern 2 is designed to capture equivalent metadata structures (Object Class, Property, Value Domain, etc.) of a CDE informed by ISO 11179. As shown in Table 2, Pattern 2 is represented in a post-coordination manner following certain rules. The approach used in Pattern 2 is similar to the dissection approach that is a common practice used in the terminology space for development of reusable terminologies. The dissection approach was originally used by the GALEN project [24]. In fact, the components in the metadata structure are usually annotated with concept codes from a standard terminology. In NCI caDSR, NCI Thesaurus has been largely used for the annotation purpose. Taking a look at Pattern 2 as shown in Table 2, “Pharmacologic Substance”, an object class, has NCIt code C1909 annotated; “Begin Occurrence”, a property, has NCI codes “C25431:C25275” annotated. In addition, the post-coordination-based approach enabled us to globally retrieve all properties associated with a particular object class. For example, there are globally 40 properties associated with the object class “Pharmacologic Substance” in NCI caDSR, resulting in additional 37 more properties and 5 more associated value domains. Figure 9 also shows such enrichment for the domain Clinical Pharmaceutical. We believe that our approach would produce a rich collection of archetype patterns and constraints (e.g., datatypes, value sets, terminology bindings, etc.) that could be used to facilitate detailed clinical modeling in clinical cancer study domain for use cases beyond TCGA.

The third implication is that we demonstrated the value of using Semantic Web technologies and tools in building such metadata repository. First, we created an OWL rendering of CIMI Reference Model. This allowed us to seamlessly integrate the CIMI Reference Model with an existing OWL-based ISO 11179 model. We envision that CIMI Reference Model and ISO 11179 are two complementary standards that could greatly enhance the detailed clinical modeling and its metadata management. Second, we used XML2RDF Transformation technology to transform the XML-based TCGA data dictionary and the XML-based caDSR CDE dataset into a RDF-based format. This allows us to use standard SPARQL query language to define queries to retrieve metadata of a CDE across datasets while this enables a high-throughput approach for globally searching metadata of nearly 50,000 CDEs recorded in the NCI caDSR. Third, we populated reusable archetype patterns against the OWL-based schema using a RDF-based representation. This will allow us to leverage the built-in OWL DL reasoning capability and the RDF validation tools such as Shape Expressions [25] to check the consistency and data quality of CIMI-compliant detailed clinical models.

## Conclusion

In summary, we developed a use case-driven approach that enables a Semantic Web-based metadata repository in support of authoring detailed clinical models in clinical cancer study domains. Future work will include 1) developing Semantic Web-based RESTful services for the archetype patterns recorded in the metadata repository; 2) building quality assurance mechanism for CIMI-compliant detailed clinical models leveraging OWL DL reasoning and RDF validation tools; 3) creating tools for authoring detailed clinical models using the metadata repository as the backend; 4) developing tools that enable the transformation of detailed clinical models between RDF/OWL-based format and ADL-based format.

## Abbreviations

ADL: Archetype definition language
BCR: Biospecimen core resource
caDSR: Cancer data standards repository
CDASH: Clinical data acquisition standards harmonization
CDISC: Clinical data interchange standards consortium
CEMS: Clinical element models
CIMI: Clinical information modeling initiative
CDEs: Common data elements
DCMs: Detailed clinical models
EHR: Electronic health record
EVS: Enterprise vocabulary services
MDR: Metadata registry
NCI: National Cancer Institute
NHGRI: National Human Genome Research Institute
ODM: Ontology definition meta-model
OMG: Object management group
ONC: Office of the National Coordinator
RDF: Resource description framework
RM: Reference model
SHARPn: Strategic health it advanced research projects area 4
TCGA: The cancer genome atlas
UML: Unified modeling language
